# MicroRNA-542-3p targets *Pten* to inhibit the myoblasts proliferation but suppresses myogenic differentiation independent of targeted *Pten*

**DOI:** 10.1186/s12864-024-10260-y

**Published:** 2024-04-01

**Authors:** Dandan Li, Yongqi Yue, Xinxin Feng, Weibing Lv, Yilin Fan, Peiran Sha, Te Zhao, Yaqiu Lin, Xianrong Xiong, Jian Li, Yan Xiong

**Affiliations:** 1 Key Laboratory of Qinghai-Tibetan Plateau Animal Genetic Resource Reservation and Utilization, Ministry of Education/Sichuan Province, Southwest Minzu University, Chengdu, 610041 China; 2 College of Animal & Veterinary Sciences, Southwest Minzu University, Chengdu, 610041 China; 3 Key Laboratory of Animal Science of National Ethnic Affairs Commission of China, Southwest Minzu University, Chengdu, 610041 China; 4 College of Animal Science and Technology, Northwest A&F University, Shaanxi, 712100 China; 5 Chongxin County Animal Husbandry and Veterinary Center, Pingliang, 744200 China

**Keywords:** miR-542-3p, Proliferation, Differentiation, *Pten*, Myoblast

## Abstract

**Background:**

Non-coding RNA is a key epigenetic regulation factor during skeletal muscle development and postnatal growth, and miR-542-3p was reported to be conserved and highly expressed in the skeletal muscle among different species. However, its exact functions in the proliferation of muscle stem cells and myogenesis remain to be determined.

**Methods:**

Transfection of proliferative and differentiated C2C12 cells used miR-542-3p mimic and inhibitor. RT-qPCR, EdU staining, immunofluorescence staining, cell counting kit 8 (CCK-8), and Western blot were used to evaluate the proliferation and myogenic differentiation caused by miR-542-3p. The dual luciferase reporter analysis and rescued experiment of the target gene were used to reveal the molecular mechanism.

**Results:**

The data shows overexpression of miR-542-3p downregulation of mRNA and protein levels of proliferation marker genes, reduction of EdU^+^ cells, and cellular vitality. Additionally, knocking it down promoted the aforementioned phenotypes. For differentiation, the miR-542-3p gain-of-function reduced both mRNA and protein levels of myogenic genes, including MYOG, MYOD1, et al. Furthermore, immunofluorescence staining immunized by MYHC antibody showed that the myotube number, fluorescence intensity, differentiation index, and myotube fusion index all decreased in the miR-542-3p mimic group, compared with the control group. Conversely, these phenotypes exhibited an increased trend in the miR-542-3p inhibitor group. Mechanistically, phosphatase and tensin homolog (*Pten*) was identified as the bona fide target gene of miR-542-3p by dual luciferase reporter gene assay, si-*Pten* combined with miR-542-3p inhibitor treatments totally rescued the promotion of proliferation by loss-function of miR-542-3p.

**Conclusions:**

This study indicates that miR-542-3p inhibits the proliferation and differentiation of myoblast and *Pten* is a dependent target gene of miR-542-3p in myoblast proliferation, but not in differentiation.

**Supplementary Information:**

The online version contains supplementary material available at 10.1186/s12864-024-10260-y.

## Background

Skeletal muscle is one of the most important organs and tissues in mammals [1], accounting for 40% of the body weight [2]. Therefore, the normal development of skeletal muscle will directly affect the life activities of the whole body, such as the energy transformation of the body to produce power, directly or indirectly participate in energy metabolism, and affect individual health [3]. Skeletal muscle development is mainly realized through the proliferation and differentiation of skeletal muscle progenitor cells and hypertrophy and thickening of skeletal muscle fibers [4]. This process is regulated by several factors, including transcription factors [5], methyltransferases [6], non-coding RNA [7], and signaling pathways [8]. However, the development process of the entire skeletal muscle is extremely complex and the key regulators are not completely revealed.

MicroRNAs (miRNAs) are a class of highly conserved endogenous non-coding RNAs with 18 to 22 nucleotides [9]. It acts by binding to the 3'-UTR (untranslated region) site of the target gene resulting in mRNA degradation or translation disruption [10]. In the process of skeletal muscle development, some muscle-specific miRNAs or muscle-rich expressions of miRNAs involved in myogenesis were reported. For example, miR-185-3p and miR-370-3p are highly and specifically expressed in leg muscles of New Zealand rabbit fetuses, children, and adults, affecting muscle development [11], and miR-22 can inhibit the proliferation of C2C12 cells and promote their differentiation [12]. More and more studies indicate that multiple muscle-enriched miRNAs play an extremely important and essential role in muscle development. Guo et al. found that miR-542-3p showed a very high average RNA level among the newly annotated miRNAs during goat skeletal muscle development, and showed a significant decrease after birth [13]. Also, previously published report found that miR-542-3p significantly increased in muscle dysfunction patients [14, 15]. Another evidence showed that miR-542-3p had a trend of first increasing and then decreasing in the porcine five prenatal stages (35, 49, 63, 77, 91 days post coitum) and five postnatal stages (2, 28, 90, 120, 180 days postnatal), which was identified as a new candidate myogenic miRNA [16]. However, the specific role and molecular mechanism of miR-542-3p in regulating the proliferation and differentiation of myoblast remain unclear.

In this study, we aim to measure the expression pattern of miR-542-3p during C2C12 myoblasts myogenic differentiation and reveal the potential role of miR-542-3p in the proliferation and differentiation of myoblasts by gain and loss of miR-542-3p function. Our results show that miR-542-3p directly targets *Pten* to negatively regulate the proliferation of myoblasts, but does not depend on *Pten* to inhibit differentiation.

## Results

### Expression patterns of miR-542-3p in different species and during myogenesis

**Fig. 1:**
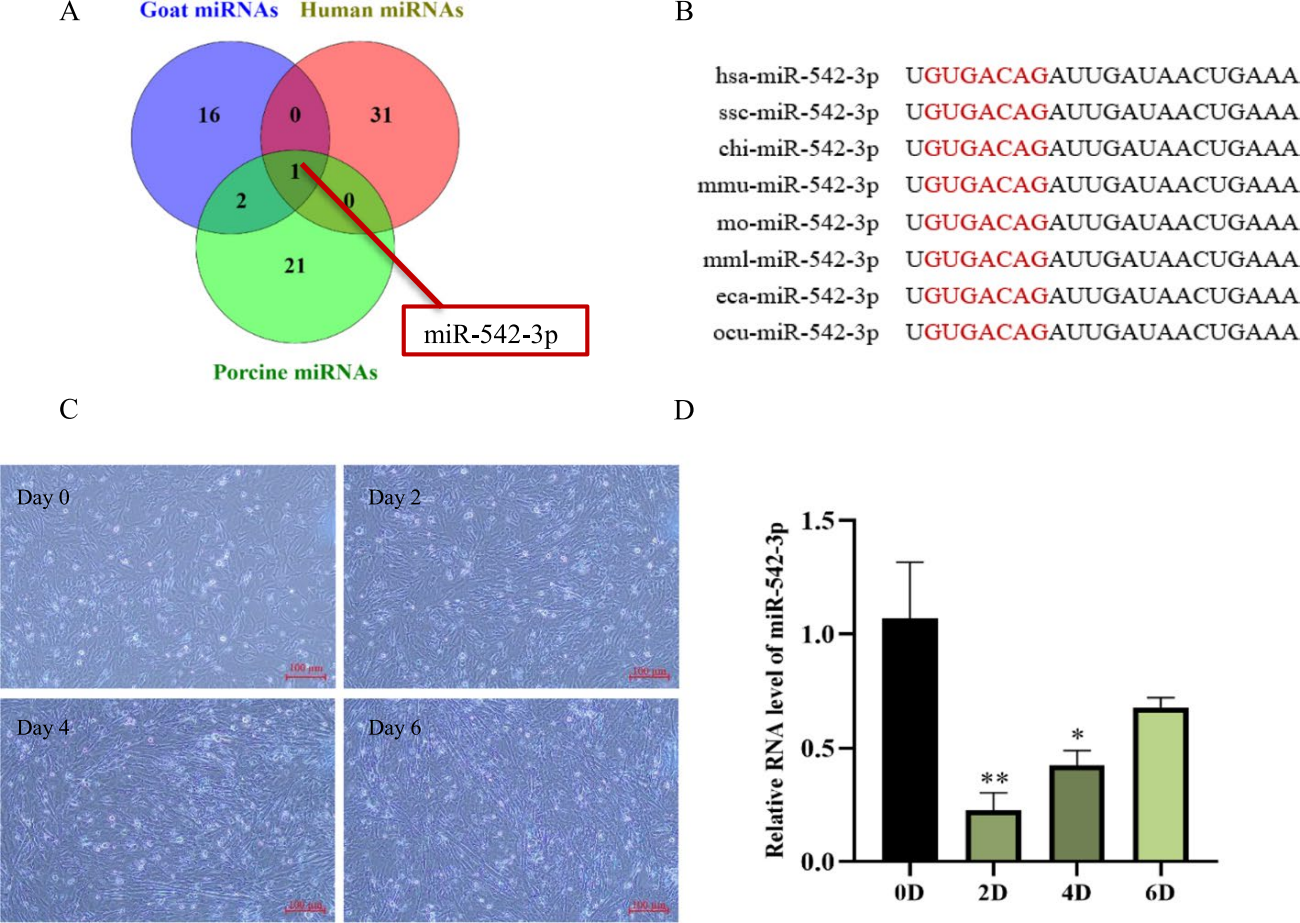
The expression pattern of miR-542-3p during myogenesis. **A** miR-542-3p was identified as a key candidate miRNA in the skeletal muscle development of goat and porcine, and muscle dysfunction patients. **B** The sequence alignment analysis of miR-542-3p in different species. The red-marked nucleotides were the seed sequence of miR-542-3p. hsa: homo sapiens; ssc: sus scrofa; chi: capra hircus; mmu: mus musculus; mo: rat; mml: macaca mulatta; eca: equus caballus; ocu: oryctolagus cuniculus. **C**, **D** The myoblasts morphology (**C**) and the expression level of miR-542-3p (**D**) at day 0, 2, 4, and 6 after myogenic differentiation, scale bar: 100 μm. *n* = 3, * P < 0.05, ** P < *0.01*

Combined analysis of the differential miRNAs in different models, including muscle dystrophy patients [15], porcine [16] and goat [13] skeletal muscle development, of which 32 differential miRNAs between male muscle dysfunction patients and healthy men of similar age were found, 24 candidate myogenic miRNAs were screened during 10 developmental stages of porcine skeletal muscle and 19 miRNAs were enriched in the goat skeletal muscle, only miR-542-3p as the overlapped miRNA in all models (Fig. 1A). The sequence alignment analysis showed that miR-542-3p was highly conserved in different species (Fig. 1B). C2C12 myoblasts were used to construct the skeletal myogenesis model (Fig. 1C), RT-qPCR results showed that miR-542-3p expression showed a sharp decreasing trend at day 2 after myogenic differentiation induction and then gradually increased during myoblast differentiation (Fig. 1D). This expressional pattern suggested that miR-542-3p might be involved in myoblast proliferation and differentiation.

### Overexpression of miR-542-3p inhibits the proliferation of myoblast

**Fig. 2:**
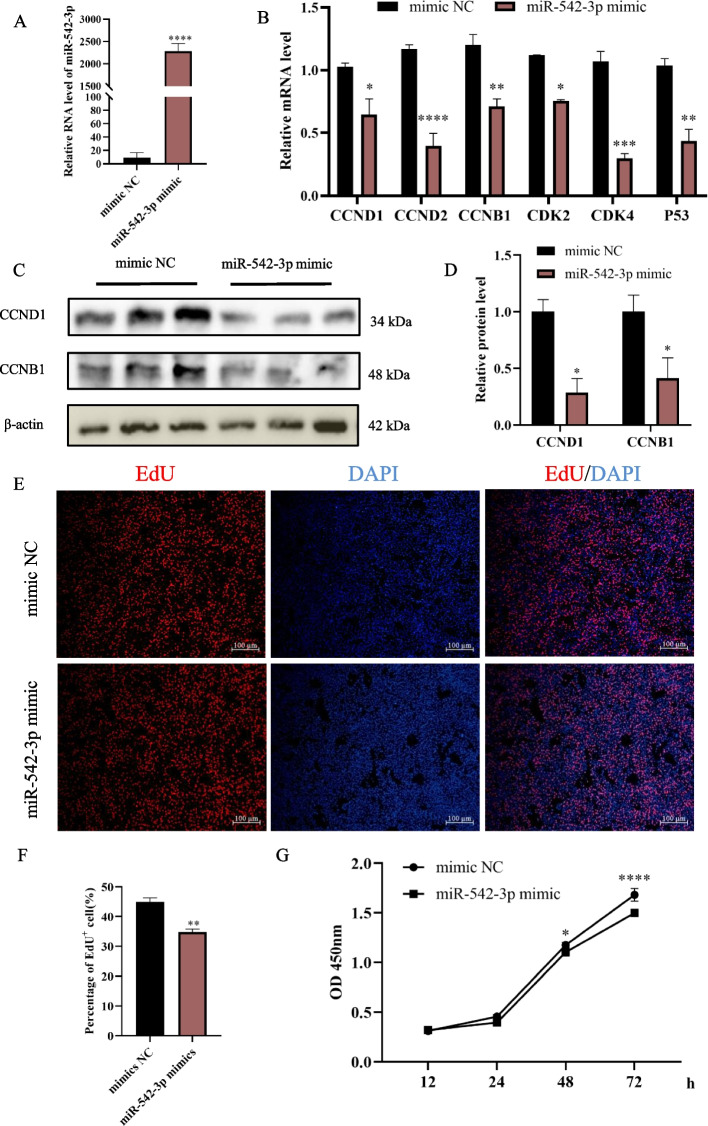
Overexpression of miR-542-3p inhibits myoblast proliferation. **A** The expression level of miR-542-3p in C2C12 myoblasts at 48 h after its mimic transfection was determined by RT-qPCR. **B** The mRNA level of *CCND1*, *CCND2*, *CCNB1*, *CDK2*, *CDK4*, and *P53* in the miR-542-3p mimic and control groups. **C**, **D** The western blotting bands of proliferation markers in the miR-542-3p mimic and control groups (**C**) and their quantitative analysis (**D**) by image J software. The membrane was cleaved before hybridization with the antibody, and all protein bands (CCND1, CCNB1, and β-actin) in Fig. 2C were from the same samples. 4 biological replicates were detected, 3 of which were shown in the Fig. 2C and full-length blots were presented in Supplementary Fig. S2. **E**,** F** The images of 5-ethynyl-20-deoxyuridine (EdU) staining (**E**) and percentage of the EdU positive cells (**F**), scale bar: 100 μm. **G** Cell viability was measured by cell counting kit-8 assay (CCK-8). *n* = 3, * P < 0.05, ** P < 0.01, *** P < 0.001, **** P < *0.0001*

Overexpression of miR-542-3p transfected by its mimic in C2C12 cells significantly downregulated the mRNA levels of proliferation marker genes, including cyclin d1 (*CCND1*), cyclin d2 (*CCND2*), cyclin b1 (*CCNB1*), cyclin dependent kinase 2 (*CDK2*), cyclin dependent kinase 4 (*CDK4*), and *P53* (Fig. 2A, B). Western blotting analysis also showed that miR-542-3p inhibited proliferation-related protein levels, CCND1 decreasing to ~ 30% and CCNB1 decreasing to ~ 40% to counterparts of control (Fig. 2C, D). Moreover, EdU staining analysis showed miR-542-3p mimic group with significant inhibition of DNA synthesis ability, compared with the control (Fig. 2E, F). In compliance with the aforementioned assays, the cell vitality also decreased significantly in the miR-542-3p mimic treated cells by CCK-8 analysis (Fig. 2G). These data indicated that overexpression of miR-542-3p inhibits the proliferation of myoblast.

### Knockdown of miR-542-3p promotes the proliferation of myoblast

**Fig. 3:**
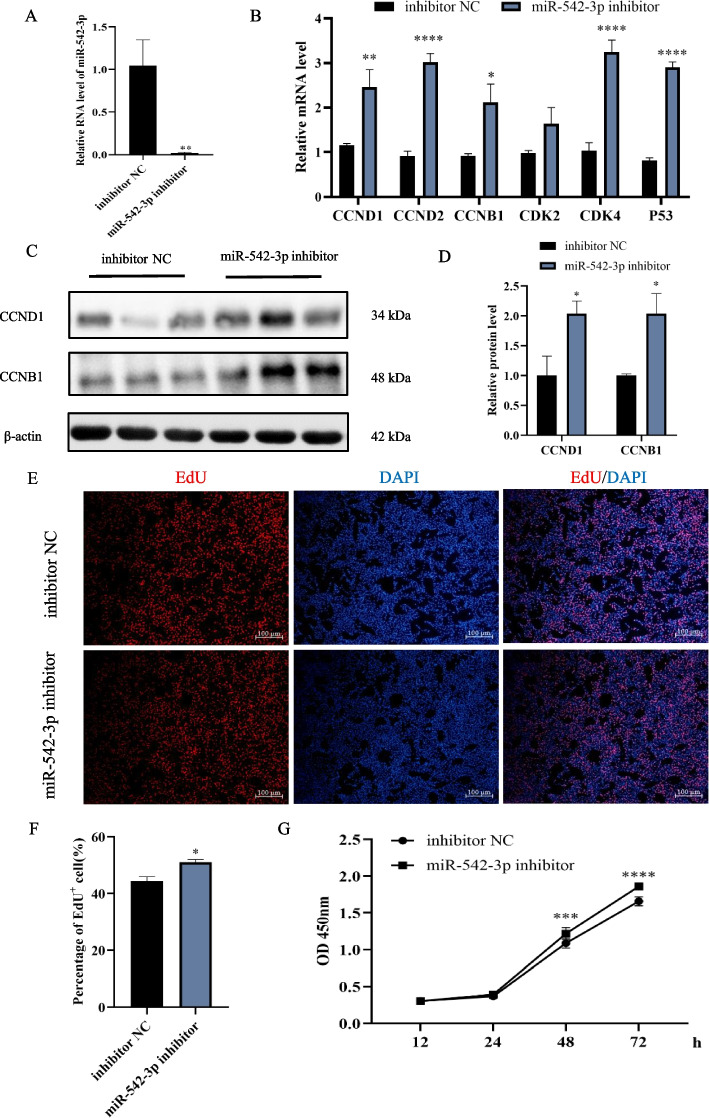
Disruption of miR-542-3p enhances the proliferation of myoblast. **A** The expression of miR-542-3p in C2C12 myoblasts at 48 h after its inhibitor treatment was assayed by RT-qPCR. **B** The mRNA levels of *CCND1*, *CCND2*, *CCNB1*, *CDK2*, *CDK4*, and *P53* in the miR-542-3p inhibitor and inhibitor NC groups. **C**,** D** The western blotting bands of proliferation markers in the miR-542-3p inhibitor and inhibitor NC groups (**C**) and their quantitative analysis (**D**) by image J software. The membrane was cleaved before hybridization with the antibody, and all protein bands (CCND1, CCNB1, and β-actin) in Fig. 3C were from the same samples. 4 biological replicates were detected, 3 of which were shown in the Fig. 3C and full-length blots were presented in Supplementary Fig. S3. **E**,** F** The images of EdU staining (**E**) and percentage of the EdU positive cells (**F**), scale bar: 100 μm. **G** Cell viability was measured by CCK-8. *n* = 3, * P < 0.05, ** P < 0.01, *** P < 0.001, **** P < *0.0001*

Interestingly, miR-542-3p inhibitor treatment dramatically suppressed its level in C2C12 myoblasts measured by RT-qPCR (Fig. 3A), which enhanced the mRNA expression of proliferation marker genes, including *CCND1* with ~ 2-, *CCND2* with ~ 3-, *CCNB1* with ~ 3-, *CDK4* with ~ 3.5-, *P53* with ~ 3-fold changes to those of control (Fig. 3B). Western blotting analysis showed that knockdown of miR-542-3p significantly upregulated protein levels of CCND1 and CCNB1 (Fig. 3C, D). Consistently, disruption of miR-542-3p increased the percentage of EdU-positive cells by EdU staining (Fig. 3E, F) and cellular vitality by CCK-8 analysis (Fig. 3G). Thus, it is concluded that miR-542-3p inhibits myoblast proliferation.

### MiR-542-3p gain-of-function inhibits the differentiation of myoblast

**Fig. 4:**
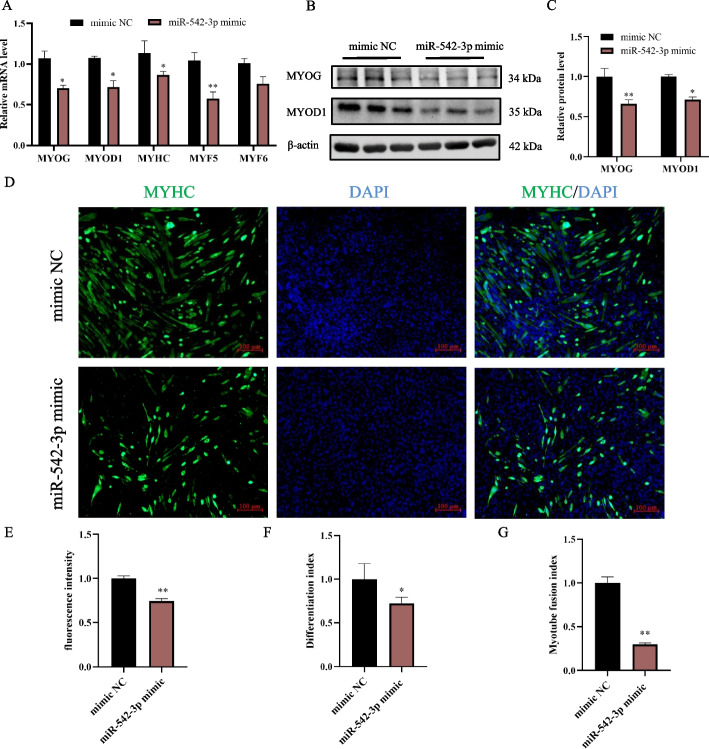
Overexpression of miR-542-3p inhibits myoblast differentiation. **A** The mRNA level of *MYOG*, *MYOD1*, *MYHC*, *MYF5*, and *MYF6* at day 3 after transfection of miR-542-3p mimic and control groups. **B**,** C** The western blotting bands of differentiation markers in the miR-542-3p mimic and control groups (**B**) and their quantitative analysis (**C**) by image J software. The membrane was cleaved before hybridization with the antibody, and all protein bands (MYOG, MYOD1, and β-actin) in Fig. 4B were from the same samples. 4 biological replicates were detected, 3 of which were shown in the Fig. 4B and full-length blots were presented in Supplementary Fig. S4. **D** Anti-Myosin heavy chain (MYHC) immunofluorescence staining after the transfection of miR-542-3p mimic, scale bar: 100 μm. **E**,** F**,** G** MYHC immunofluorescence intensity analysis (**E**), differentiation index (**F**), myotube fusion index (**G**) by image J software. *n* = 3, * P < 0.05, ** P < *0.01*

Previous results showed that the expression of miR-542-3p decreased at the early differentiation stage and then gradually increased during myoblast differentiation (Fig. 1D), which suggested miR-542-3p might participate in myogenic differentiation. Overexpression of miR-542-3p was performed and data showed that all the myogenic marker genes, including myogenin (*MYOG*), myogenic differentiation 1 (*MYOD1*), myosin heavy chain (*MYHC*), myogenic factor 5 (*MYF5*) and myogenic factor 6 (*MYF6*), were inhibited in the miR-542-3p mimic groups (Fig. 4A). Consistent with the transcriptional expression, miR-542-3p mimic treatment had significantly lower protein levels of MYOG and MYOD1 than those of mimic NC by western blotting analysis (Fig. 4B, C). Immunofluorescence staining immunized by MYHC antibody showed overexpression of miR-542-3p with fewer and smaller myotubes (Fig. 4D), indicated by the significant reduction of fluorescence intensity (Fig. 4E), differentiation index (Fig. 4F), and myotube fusion index (Fig. 4G). Therefore, miR-542-3p gain-of-function inhibits the differentiation of myoblast.

### MiR-542-3p loss-of-function enhances the differentiation of myoblast

**Fig. 5:**
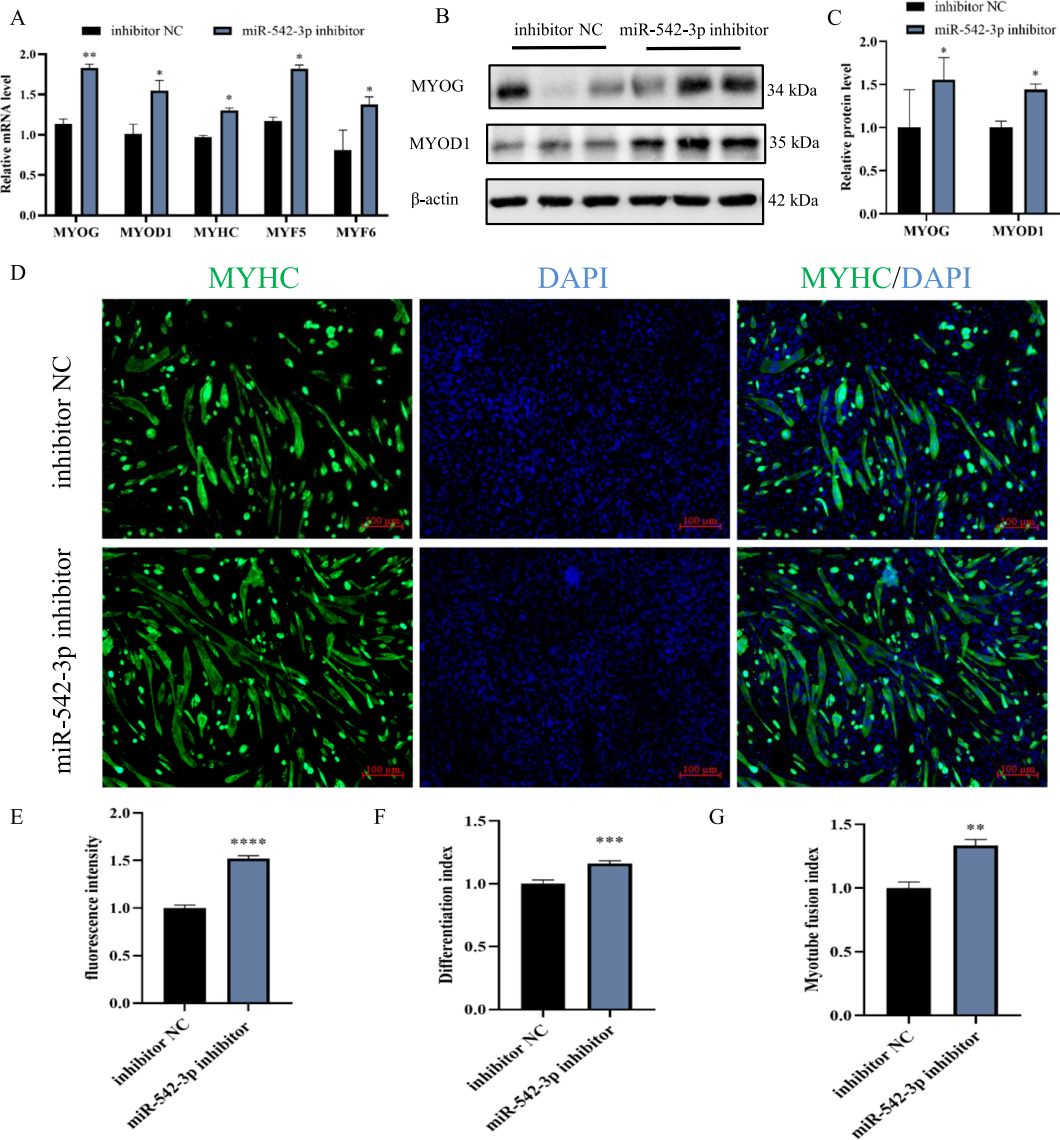
Inhibition of miR-542-3p promotes myoblast differentiation. **A** The mRNA improvement of *MYOG*, *MYOD1*, *MYHC*, *MYF5*, and *MYF6* at day 3 after transfection of miR-542-3p inhibitor and control groups. **B**,** C** The western blotting bands of differentiation markers in the miR-542-3p inhibitor and control groups (**B**) and their quantitative (**C**) analysis by image J software. The membrane was cleaved before hybridization with the antibody, and all protein bands (MYOG, MYOD1, and β-actin) in Fig. 5B were from the same samples. 4 biological replicates were detected, 3 of which were shown in the Fig. 5B and full-length blots were presented in Supplementary Fig. S5. **D** Anti-Myosin heavy chain (MYHC) immunofluorescence staining after the transfection of miR-542-3p inhibitor, scale bar: 100 μm. **E**, **F**,** G** MYHC immunofluorescence intensity analysis (**E**), differentiation index (**F**) and myotube fusion index (**G**) by image J software. *n* = 3, * P < 0.05, ** P < 0.01, *** P < 0.001, **** P < *0.0001*

Next, we carried out miR-542-3p loss-of-function in C2C12 cells, which significantly increased the gene expression levels of *MYOG*, *MYOD1*, *MYF5*, and *MYF6*, compared to those of control (Fig. 5A). Western blotting result showed that interference of miR-542-3p dramatically upregulated protein levels of MYOG and MYOD1 (Fig. 5B, C). In compliance with the gene expression, disruption of miR-542-3p had more and larger myotubes labeled by MYHC antibody (Fig. 5D) and quantitative analysis found a significant increase trend of fluorescence intensity (Fig. 5E), differentiation index (Fig. 5F), and myotube fusion index (Fig. 5G) in miR-542-3p inhibitor-treated cells to counterparts of control. Therefore, we infer that inhibition of miR-542-3p enhances myoblast differentiation.

### *Pten* is identified as a direct target gene of miR-542-3p

**Fig. 6:**
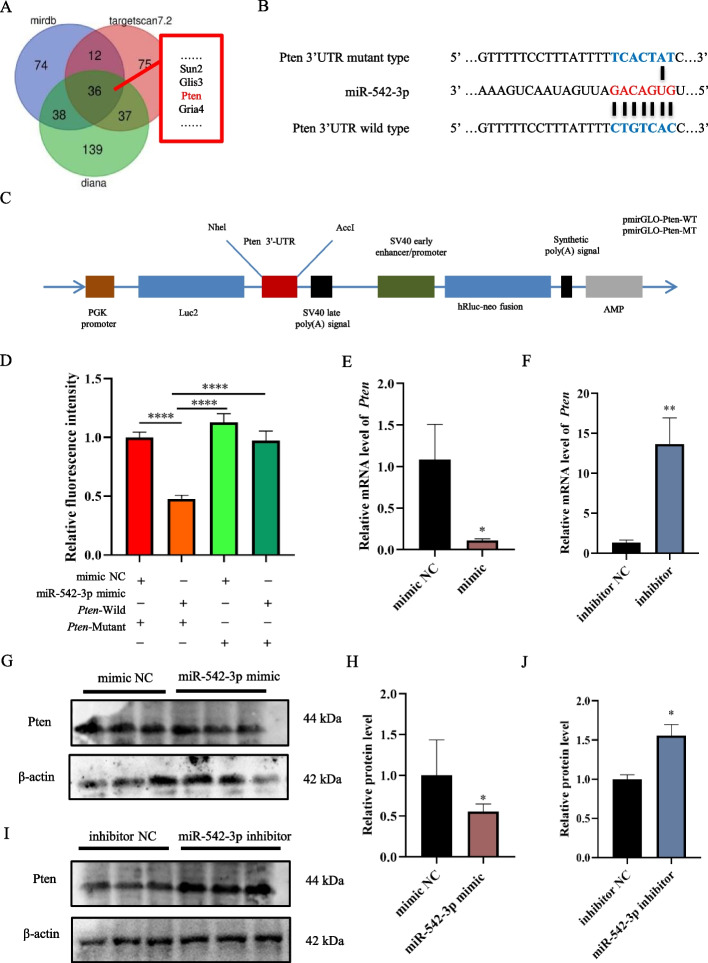
miR-542-3p directly targets the *Pten* gene. **A** Prediction of target genes of miR-542-3p using online software, including DIANA, TargetScan 7.2, and miRDB. **B** The complementary pairing of miR-542-3p and the potential target gene *Pten* wild type 3’-UTR or its mutated 3’-UTR. **C** Diagram of the construction of dual-luciferase reporter vectors containing the *Pten* 3’-UTR sequences of wild type or mutant. hRluc-neo fusion means renilla luciferase; Luc2 means firefly luciferase. **D** 293 T cells were co-transfected with *Pten*-3’-UTR wild or mutant dual-luciferase vector and the miR-542-3p mimic or mimic NC. The relative luciferase activity was assayed at 48 h after transfection. **E**,** F** The mRNA level of *Pten* in the miR-542-3p mimic (mimic) and mimic NC (**E**), miR-542-3p inhibitor (inhibitor) and inhibitor NC (**F**) were determined by RT-qPCR. **G**,** I**,** H**,** J** Protein level of Pten was detected by western blotting after overexpression (**G**,** H**) and inhibition (**I**,** J**) of miR-542-3p. The membrane was cleaved before hybridization with the antibody, and all protein bands in Fig. 6G and protein bands in Fig. 6I were from the same sample, respectively. Full-length blots are presented in Supplementary Fig. S6. *n* = 3, * P < 0.05, ** P < *0.01*

To further investigate the bona fide target of miR-542-3p in myoblast proliferation and differentiation, three online software tools, including Targetscan 7.2, miRDB, and DIANA, were used to predict the target gene of miR-542-3p. The Venn diagrams showed 36 overlapping target genes, such as *Sun2*, *Glis3*, *Gria4*, and *Pten* (Fig. 6A). Numerous studies have shown that *Pten* has an important effect on the growth and development of skeletal muscle [17–19]. Therefore, *Pten* was predicted as a candidate target gene of miR-542-3p in the skeletal muscle. The dual luciferase reporter plasmids (PmirGLO-*Pten*-WT and PmirGLO-*Pten*-MT) were constructed, in which pmirGLO-*Pten*-WT was inserted by the wild type 3'-UTR sequence of *Pten* binding to seed sequence of miR-542-3p and PmirGLO-*Pten*-MT was inserted by *Pten* 3'-UTR with nucleotide mutation at binding site (Fig. 6B, C and Supplementary Material Fig. 1). Next, PmirGLO-*Pten*-WT, PmirGLO-*Pten*-MT, miR-542-3p mimic, and mimic NC were co-transfected to perform luciferase reporter assay and data showed that miR-542-3p mimic treatment dramatically suppressed luciferase activity of PmirGLO-*Pten*-WT, compared to mimic NC group (Fig. 6D). In contrast, luciferase activity of PmirGLO-*Pten*-MT was not affected by miR-542-3p mimic (Fig. 6D). Furthermore, overexpression of miR-542-3p inhibited *Pten* expression at mRNA levels (Fig. 6E), while knockdown of miR-542-3p elevated its mRNA levels (Fig. 6F). In compliance with transcriptional level, the inhibition and elevation of Pten protein level were respectively observed in miR-542-3p mimic and its inhibitor treatment cells by western blotting analysis (Fig. 6G, H, I, J). Thus, these results indicated that *Pten* is a direct target gene for miR-542-3p.

### MiR-542-3p inhibits myoblasts proliferation by targeting *Pten*

**Fig. 7:**
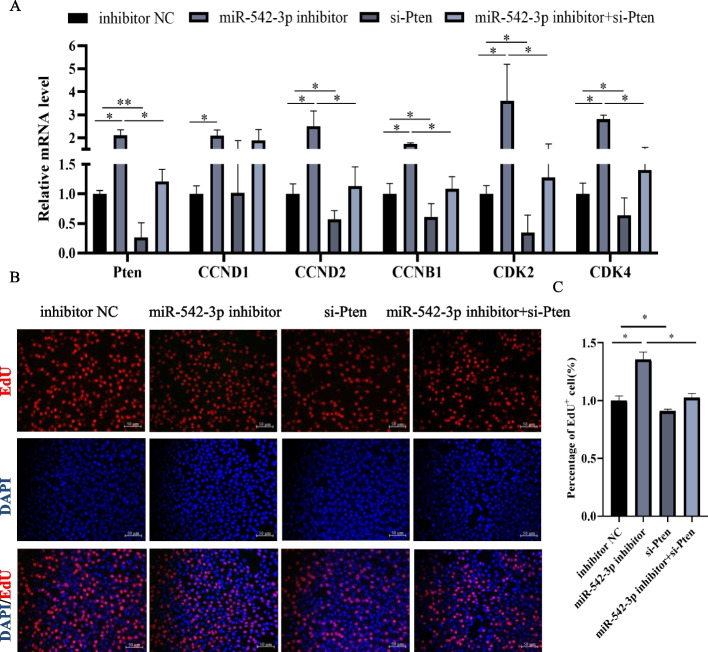
miR-542-3p inhibits myoblasts proliferation through targeting *Pten*. **A** The mRNA level of *Pten*, *CCND1*, *CCND2*, *CCNB1*, *CDK2*, and *CDK4* in the miR-542-3p inhibitor, si-*Pten*, miR-542-3p inhibitor + si-*Pten* and control groups. **B**,**C** The images of 5-ethynyl-2'-deoxyuridine (EdU) staining (**B**) and percentage of the EdU positive cells (**C**), scale bar: 50 μm. *n* = 3, * P < 0.05, ** P < *0.01*

Shen et al*.* found that *Pten* positively regulates the proliferation of skeletal muscle satellite cell [20]. Firstly, the function of *Pten* regulation on myoblast proliferation was confirmed mediated by si-*Pten* treatment. RT-qPCR results showed that the knockdown efficiency of *Pten* was high and decreased to ~ 20% to that of control (Fig. 7A), which significantly downregulated mRNA levels of proliferation-related marker genes, including *CCNB1*, *CCND2*, *CDK2*, and *CDK4* (Fig. 7A). Moreover, knockdown of *Pten* exhibited an obvious reduction of EdU positive cells (Fig. 7B, C). These data were concluded that the knockdown of *Pten* suppressed myoblast proliferation.

Then, the rescue experiments of co-transfected si-*Pten* and miR-542-3p inhibitor were carried out to validate miR-542-3p inhibiting myoblasts proliferation depending on target gene *Pten*. Compared with inhibitor NC and miR-542-3p inhibitor groups, miR-542-3p inhibitor combination with the si-*Pten* group totally rescued the mRNA level of proliferation-related genes (Fig. 7A). Similarly, the DNA synthesis ability of myoblast, another indicator of cellular proliferation, assessed by EdU staining analysis was identical to the expression of proliferated related genes (Fig. 7B, C). These results further demonstrated that miR-542-3p inhibits the proliferation of C2C12 myoblasts by directly targeting *Pten*.

### MiR-542-3p inhibits myoblasts differentiation independent of targeted *Pten*

**Fig. 8:**
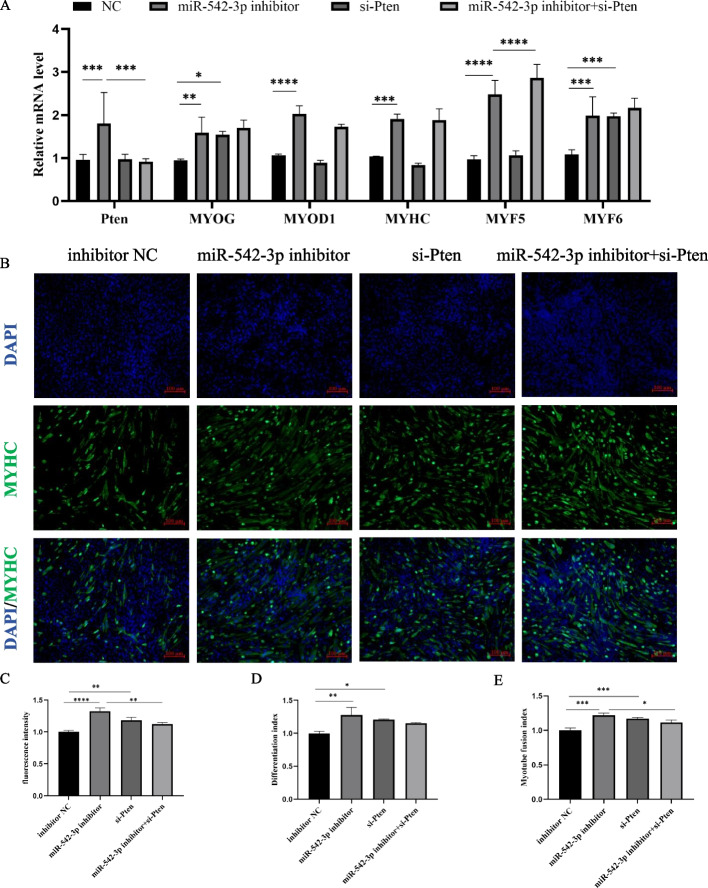
miR-542-3p inhibits myoblasts differentiation. **A** The mRNA level of *Pten*, *MYOG*, *MYOD1*, *MYHC*, *MYF5*, and *MYF6* in the miR-542-3p inhibitor, si-*Pten*, miR-542-3p inhibitor + si-*Pten*, and control groups were detected by RT-qPCR. **B** Anti-Myosin heavy chain (MYHC) immunofluorescence staining after the transfection of miR-542-3p inhibitor, si-*Pten*, miR-542-3p inhibitor + si-*Pten*, and control groups. DAPI (blue), cell nuclei, scale bar: 100 μm. **C** MYHC immunofluorescence intensity analysis by image J software. **D** Differentiation index. **E** Myotube fusion index. *n* = 3, * P < 0.05; ** P < 0.01; *** P < 0.001; **** P < *0.0001*

Yue et al*.* found that the skeletal muscle-specific knockout (KO) *Pten* gene can alleviate muscle fiber degeneration, restore muscle function, and improve muscle pathology [19]. To validate the effect of *Pten* on the differentiation of C2C12 myoblasts, si-*Pten* and negative control (NC) were transfected, respectively. RT-qPCR results showed that knockdown of *Pten* significantly promoted the mRNA levels of the differentiated related marker genes *MYOG* and *MYF6* were also significantly increased (Fig. 8A). In addition, MYHC antibody immunofluorescence staining found more myotubes in the si-*Pten* group than that of inhibitor NC (Fig. 8B) and had a significant increase trend in the fluorescence intensity (Fig. 8C), differentiation index (Fig. 8D), and myotube fusion index (Fig. 8E) of the si-*Pten* group by quantitative analysis. These data indicated that the knockdown of *Pten* promotes myoblast differentiation.

**Fig. 9:**
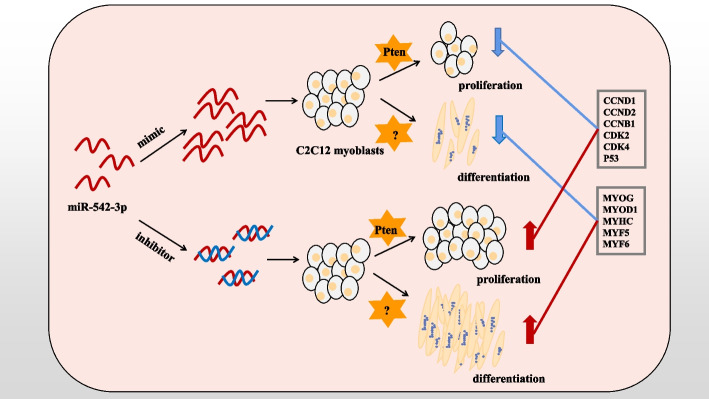
Schematic diagram of miR-542-3p regulation on myoblasts proliferation and differentiation

Then, we carried out the rescue experiments of co-transfected si-*Pten* and miR-542-3p inhibitor to elucidate miR-542-3p repressing myogenic differentiation through the target gene *Pten*. It was found that the differentiation-associated genes had no significant change except for *MYF5*, compared with the miR-542-3p inhibitor group, so the promotion of myogenic differentiation induced by the miR-542-3p inhibitor was not rescued (Fig. 8A). Further, myotubes formation, differentiation index, and myotube fusion index were determined by immunofluorescence staining and its quantitative analysis, which was also identical to gene expressional trend (Fig. 8B, C, D, E). In summary, miR-542-3p inhibits the differentiation of myoblasts independently of targeting *Pten*. These results demonstrated that miR-542-3p inhibits myoblast differentiation and relies on the target gene *Pten* to inhibit proliferation (Fig. 9).

## Discussion

More and more pieces of evidence indicate that multiple muscle-enriched miRNAs play an extremely important and essential role in muscle development. Here, overexpression of miR-542-3p inhibits the proliferation and differentiation of myoblast. On the contrary, the knockdown of miR-542-3p promotes these phenotypes. This study proves that miR-542-3p is a negative regulator of myoblast proliferation and myogenic differentiation.

Extensive research demonstrated that miRNAs are highly conserved in the minimal [21–23]. Through literature and sequence alignment analysis, miR-542-3p is the only highly conserved difference miRNA in skeletal muscle development of goats [13], muscular dystrophy patients [15] and porcine [16]. Therefore, miR-542-3p was identified as a conserved in different species. Besides, we found that the expression of miR-542-3p decreased sharply after 2 days of induction, and then gradually increased. Research has shown that miR-542-3p has a trend of first increasing and then decreasing in the five prenatal and postnatal stages of porcine [16] and plays an important role in skeletal muscle of yaks aged 0.5, 2.5, 4.5, and 7.5 years [24]. Therefore, miR-542-3p might participate in the proliferation and differentiation of myoblasts.

*CCNB1* [25], *CDK2* [26], *CCND1* [27], *CCND2* [28], *CDK4* [29], and *P53* [30] are all marker genes associated with cell proliferation, and our data show that overexpression of miR-542-3p significantly inhibits these gene expression, cellular activity and DNA synthesis and vice versa. These data indicate that miR-542-3p inhibits the proliferation of C2C12 myoblasts. Similarly, extensive studies have shown that miR-542-3p has an inhibitory effect on the proliferation of cancer cells, including colorectal cancer cell [31], neuroblastoma cell [32], and epithelial ovarian cancer [33]. Based on previously published work, Qin et al. found that the expression of miR-542-3p in porcine peaked at 63 days after coitum and gradually decreased later [16], and Guo et al. found that miR-542-3p peaked at 60 days of gestation and decreased significantly at 3 days after birth in the four developmental stages of longissimus dorsi muscle of goats [13]. These results suggested that miR-542-3p is highly expressed during the fetal period (a high cellular proliferation phase) of porcine and goat, which suggested that miR-542-3p might be a positive regulator for muscle stem cell proliferation. However, overexpression of miR-542-3p inhibits C2C12 cell proliferation in vitro, and the opposite phenotype was observed after inhibition of miR-542-3p. Except for our data and the aforementioned cited works, other papers also shown that miR-542-3p gain-of-function inhibits the proliferation of cells, such as osteoblast cell [34] and breast cancer cell [35]. Combined with our data and published works, miR-542-3p is a conserved negative regulator for cell proliferation.

Overexpression and interference of miR-542-3p, the mRNA level of *P53* respectively decreased and increased along with the proliferation marker genes. However, the *P53* expressional trend mediated by miR-542-3p in our study was contradictory with its classical function, as a tumor suppressor factor [36]. It was found that P53 has a low concentration in normal cells and is transcriptionally activated after being stimulated by external factors, and its role is related to protein modifications, including ubiquitination and acetylation [37, 38]. Thus, the changes in the mRNA level of *P53* were detected, it was speculated that *P53* without modification may not play a biological function in this experiment. In addition, Roser Farre-Garros et al. reported that miR-542-3p/5p was positively correlated with *P53* activity in patients with chronic obstructive pulmonary disease (COPD), accompanied by a reduction of muscle mass and function [15]. In this case, the data just showed that the miR-542 level has positively correlated with the gene set for *P53* activity, being involved in mitochondrial ribosomal stress [15]. The miR-542 weather affects the *P53* expression or its protein modification still needs further study.

More and more miRNAs play a role in the differentiation of skeletal muscle myogenesis, such as muscle-specific miRNAs (including miR-208 [39, 40] and miR-499 [41]) and non-muscle-specific miRNAs (including miR-24, miR-122, miR-26a, miR-27b, miR-29, miR-214, miR-486, miR-499, miR-125b, etc.) [42–49]. In recent years, there has been limited literature on miR-542-3p in studying skeletal muscle development. Kureel J et al. have explained the mechanism of action of miR-542-3p in osteoblast differentiation and bone formation, and miR-542-3p acts on bone morphogenetic protein 7 (BMP-7) to inhibit osteoblast differentiation [34]. Consistently, we found that overexpression of miR-542-3p inhibited differentiation of myoblasts.

We used three online software tools (Targetscan 7.2, miRDB, and DIANA) combined with Venn diagram analysis to predict that *Pten* is a candidate target gene of miR-542-3p. Previously, researchers reported that *Pten* was targeted by miR-19b-3p, resulting in an increase of muscle protein synthesis rate in aging mice and human myotubes [50]. Additionally, Pten was discovered in 1997 independently by three laboratories as a tumor suppressor of which the expression is often lost in tumors [51–53], and several miRNAs (including miR-205, miR-122, miR-21, etc.) have been identified as binding to the 3' untranslated region of *Pten* mRNA [54–56]. Therefore, we hypothesized that miR-542-3p might inhibit myoblast proliferation and differentiation targeting *Pten*. Indeed, dual-luciferase reporter analysis and expression measurement supported that *Pten* was a *bona fide* target gene of miR-542-3p. Furthermore, the rescued experiment provided direct evidence that miR-542-3p suppresses myoblast proliferation through target *Pten*. However, the rescued phenomenon was not observed in myoblast differentiation, which suggested that the inhibition of miR-542-3p on myogenic differentiation independent of targeted *Pten* and miR-542-3p would target other genes to repress myogenesis.

## Conclusions

Collectively, miR-542-3p repressed the proliferation and differentiation of C2C12 myoblasts. Furthermore, *Pten* was identified as the downstream target gene of miR-542-3p, and its inhibition for myoblast proliferation depends on *Pten*. Our findings are conducive to understanding the specific mode of action of miR-542-3p in regulating myoblast proliferation and differentiation, which also expands knowledge of studying skeletal muscle at the miRNA level.

## Methods

### Cell culture

The C2C12 myoblasts cell line (ATCC, USA) was cultured in a growth medium consisting of Dulbecco's modified eagle medium (DMEM) (Gibco, C11995500BT, China), 10% fetal bovine serum (FBS) (AlphaCell, 100,061, China), and 1% penicillin–streptomycin (PS) (Biosharp, BL505A, Hefei, China). The cells with 80%-90% confluences were incubated in a differentiation medium (DM) containing 2% horse serum (Solarbio, S9050, Beijing, China), 1% PS, and DMEM. Cells were cultured in a cell culture incubator at constant temperature and humidity (37 °C, 5% CO_2_), and the medium was changed every 2 days.

## Cell transfection

miR-542-3p mimic, mimic negative control (NC), miR-542-3p inhibitor, and inhibitor NC were synthesized by Jima Pharmaceutical Technology (Shanghai, China) (Table 1). The above RNAs, *Pten*-wild (ligated the 3’-UTR of *Pten*) and pmir-GLO plasmids were transfected by Lipofectamine™ 3000 Reagent (Invitrogen, L3000015, USA), according to the instructions of the manufacturer. The cells were seeded in 24-well plates and the transfection was performed at 40% and 60% of cell confluence for proliferation and differentiation assay, respectively. Collected cells 48 h after transfection to detect proliferation-related indicators. To detect differentiation-related indicators, myogenic induction was performed on cells 24 h after transfection, and then cells were collected for further myogenic analysis at 3 days after induction. The miR-542-3p mimic was transfected at a concentration of 80 nmol/l (nM) and the inhibitor was transfected at a concentration of 100 nM. And the transfection concentration of *Pten*'s siRNA is 100 nM.

**Table 1:** RNA oligonucleotides for miR-542-3p and siRNA for *Pten* in this study

miRNA Name	Sequence (5’-3’)
miR-542-3p mimic	UGUGACAGAUUGAUAACUGAAA UCAGUUAUCAAUCUGUCACAUU
mimic NC	UUCUCCGAACGUGUCACGUTT ACGUGACACGUUCGGAGAATT
miR-542-3p inhibitor	UUUCAGUUAUCAAUCUGUCACA
inhibitor NC	CAGUACUUUUGUGUAGUACAA
si-Pten	GAAGUAAGGACCAGAGACAUU UGUCUCUGGUCCUUACUUCUU
Pten NC	AUUCUAUCACUAGCGUGACUU GUCACGCUAGUGAUAGAAUUU

### Total RNA extraction and quantitative real-time PCR (RT-qPCR)

TRiZOL (Vazyme, R401-01, Nanjing, China) reagent was used to extract total RNA from C2C12 myoblasts. The quality and concentration of RNA were measured by Nanodrop ND-1000 (Thermo Fisher, USA), and the OD_260_/OD_280_ between 1.8 ~ 2.0 was used to synthesize cDNA. According to the manufacturer’s instructions, Prime-Script RT Master Mix (Takara, Dalian, China) was used to synthesize cDNA. SYBR qPCR Master Mix (Vazyme, Nanjing, China) and SYBR real-time PCR mixture (BioTeke, Beijing, China) were used for real-time quantitative PCR analysis on the Bio-Rad CFX96 system. The primers used were shown in Table 2. Each sample was repeated three times and the 2-^△△Ct^ method was used to analyze data.

**Table 2:** Primer sequences for a reverse transcription-quantitative polymerase chain reaction

Gene	Forward	Reverse
miR-542-3p	5’-GCGCGTGTGACAGATTGATAA-3’	5’-AGTGCAGGGTCCGAGGTATT-3’
miR-542-3p Stem loop sequence	5’-GTCGTATCCAGTGCAGGGTCCGAGGTATTCGCACTGGATACGACTTTCAG-3’	
CCNB1	5’-CTTGCAGTGAGTGACGTAGAC-3’	5’-CCAGTTGTCGGAGATAAGCATAG-3’
CDK2	5’-CAAAGCCAAGCACGTAGAGAC-3’	5’-TGCACCACATATTGACTGTCC-3’
CDK4	5’-CTGAACCGCTTTGGCAAGAC-3’	5’-GCCCTCTCTTATCGCCAGAT-3’
CCND1	5’-TAGGCCCTCAGCCTCACTC -3’	5’-CCACCCCTGGGATAAAGCAC-3’
CCND2	5’-GAGTGGGAACTGGTAGTGTTG-3’	5’-CGCACAGAGCGATGAAGGT-3’
P53	5’-CAACAAATGCTGGCTACTAAGGA-3’	5’-CACGAGTTTTCCGTTGCTCA-3’
MYOG	5’-GAGACATCCCCCTATTTCTACCA-3’	5’-GCTCAGTCCGCTCATAGCC-3’
MYOD1	5’-CCACTCCGGGACATAGACTTG-3’	5’-AAAAGCGCAGGTCTGGTGAG-3’
MYHC	5’-GCGAATCGAGGCTCAGAACAA-3’	5’-GTAGTTCCGCCTTCGGTCTTG-3’
MYF5	5’-AAGGCTCCTGTATCCCCTCAC-3’	5’-TGACCTTCTTCAGGCGTCTAC-3’
MYF6	5’-AGAGGGCTCTCCTTTGTATCC-3’	5’-CTGCTTTCCGACGATCTGTGG-3’
U6	5’-CTCGCTTCGGCAGCACA-3’	5’-AACGCTTCACGAATTTGCGT-3’
18S	5’-CGCGGTTCTATTTTGTTGGT-3’	5’-AGTCGGCATCGTTTATGGTC-3’
RPL7	5’-ACGGTGGAGCCTTATGTGAC-3’	5’-TCCGTCAGAGGGACTGTCTT-3’
Pten	5’-TGGATTCGACTTAGACTTGACCT-3’	5’-GCGGTGTCATAATGTCTCTCAG-3’

### EdU staining

For this measurement, 10 µM 5-ethynyl -2'-deoxyuridine (Beyotime, C0078S, Shanghai, China) was added to GM and incubated for 2 h. The procedures of fixation, osmosis, and Edu staining were conducted under the manufacturer’s instruction. DAPI was used to conduct nuclear staining for 10 min. Finally, images were captured by Zeiss confocal laser microscope and the positive rate was representative of EdU positive cells/DAPI cells.

### Cell counting kit 8 (CCK-8) assay

C2C12 myoblasts were seeded in 96-well plates with 100 μl growth medium (GM) and every plate had 5 × 10^3^ cells. Negative control, mimic, and inhibitor were transfected into cells and a Cell Counting Kit 8 (AbMole, M4839, Shanghai, China) was used to detect the cell proliferative activity. In brief, 10 μl CCK-8 reagent was added into cells in every plate and a full band microplate reader (Perkin Elmer) was used to detect absorbance (450 nm) at 12 h, 24 h, 48 h, and 72 h. There are 6 biological repetitions in each group.

### Immunofluorescence staining

The cells were washed by PBS twice and 4% paraformaldehyde (Biosharp, BL539A, China) solution was added to fix cells for 10 ~ 15 min at room temperature, and then PBS was used to wash samples three times. Subsequently, 100 nM glycine was added to incubate for 10 min, and then PBS was added to wash samples 3 times. Blocking buffer (5% goat serum, 2%BSA, 0.2%TritonX-100, and 0.1% sodium azide) was used to incubate cells for 30 min at room temperature, and then cells were immunized with primary antibodies (MYHC, DSHB Hybridoma Product MF 20) for overnight at 4℃. After that, the fluorescently labeled secondary antibody diluted with PBS was added to incubate for 1 h at room temperature. The fluorescently labeled secondary antibody included Alexa Fluor™ 568 goat anti-rabbit IgG (H + L) (Invitrogen, A11011, Carlsbad, USA) and Goat anti-mouse IgG H&L/FITC (Bioss, bs-0296G-FITC, Beijing, China). The confocal microscope (Zeiss, LSM800, Germany) was used to capture images. Used ImageJ software to calculate fluorescence intensity and collected all data from randomly selected areas of 3 biological replicates. MYHC immunofluorescence intensity analysis is expressed as total fluorescence intensity divided by area. The myotube differentiation index was expressed as the ratio of the number of myotubes with 3 or more nuclei to the total number of myotubes [57]. The Myotube fusion index was expressed as the ratio of the number of nuclei in the myotube to the total number [58].

### Dual-Luciferase reporter assay

HEK293T (293 T) cells were seeded in 24-well plates and cultured in a growth medium (GM). The miR-542-3p mimic, mimic NC, and luciferase reporter plasmids with wild type and mutant 3’-UTR of *Pten* were co-transfected at 70%-80% of cell density. There are 3 repetitions in each group. The Dual-Luciferase Reporter Assay Kit (Vazyme, DL101-01, Nanjing, China) was used to detect firefly and vanilla luciferase activity, according to the manufacturer’s instructions.

### Western blot analysis

Cells were washed twice with PBS solution and lysed in RIPA protein lysis buffer with 1% protease inhibitors to extract total protein. Western blotting was performed according to a previously established procedure, and protein concentration determination was performed using a BCA protein concentration assay kit (Biosharp, BL521B, Hefei, China), followed by protein separation in SDS-PAGE, and then transferred to PVDF membrane with anti-Pten (Bioss, bsm-33319M, Beijing, China), β-actin (Bioss, bsm-33036M, Beijing, China), CCND1 (Wanleibio, WL01435a, Shenyang, China),

CCNB1 (Wanleibio, WL01760, Shenyang, China), MYOG (Abcam, ab124800, Cambridge, United Kingdom), and MYOD1 (Wanleibio, WL04662, Shenyang, China) were immunoblotted. Used ImageJ software to analyze the grayscale values of protein bands and standardize the target protein bands to β-actin was used as the load control. Each experiment was repeated with 3 organisms.

### Bioinformatic analysis

A prediction of the miRNA target gene was performed by miRDB (http://mirdb.org/), Targets can (http://www.targetscan.org/mmu_72/), and DIANA (http://diana.imis.athenainnovation.gr/DianaTools/index.php?r=microT_CDS/index). Venn Diagram was finished by the website(http://bioinformatics.psb.ugent.be/webtools/Venn/).

### Statistical analysis

Data were represented as mean ± standard error. GraphPad Prism 8.0 software and image J 1.8.0 were used to analyze data. Calculate statistically significant differences through T-test analysis and two-way ANOVA. *P* < *0.05*, the difference is significant.

### Supplementary Information

**Supplementary Material 1.**

**Supplementary Material 2.**

## Data Availability

The raw data that supports the findings of this study are available from the corresponding author upon reasonable request. The raw data was available at https://www.scidb.cn/en/s/7FjeA3.

## Abbreviations

Pten: Phosphatase and tensin homolog
miRNA: MicroRNA
CCND1: Cyclin D1
CCND2: Cyclin D2
CCNB1: Cyclin B1
CDK2: Cyclin dependent kinase 2
CDK4: Cyclin dependent kinase 4
MYOG: Myogenin
MYOD1: Myogenic differentiation 1
MYHC: Myosin heavy chain
MYF5: Myogenic factor 5
MYF6: Myogenic factor 6
EdU: 5-Ethynyl-2’-deoxyuridine
DAPI: 4’,6-Diamidino-2-phenylindole
DMEM: Dulbecco’s modified Eagle medium
RT-qPCR: Real-time quantitative PCR
NC: Negative control

## References

[1] Bjorkman KK, Guess MG, Harrison BC, Polmear MM, Peter AK, Leinwand LA (2020). miR-206 enforces a slow muscle phenotype. J Cell Sci.

[2] Brooks SV (2003). Current topics for teaching skeletal muscle physiology. Adv Physiol Educ.

[3] Frontera WR, Ochala J (2015). Skeletal muscle: a brief review of structure and function. Calcif Tissue Int.

[4] Chal J, Pourquie O (2017). Making muscle: skeletal myogenesis in vivo and in vitro. Development.

[5] Schiaffino S, Dyar KA, Ciciliot S, Blaauw B, Sandri M (2013). Mechanisms regulating skeletal muscle growth and atrophy. FEBS J.

[6] Fan Y, Liang Y, Deng K (2020). Analysis of DNA methylation profiles during sheep skeletal muscle development using whole-genome bisulfite sequencing. BMC Genomics.

[7] Guller I, Russell AP (2010). MicroRNAs in skeletal muscle: their role and regulation in development, disease and function. J Physiol.

[8] Charge SB, Rudnicki MA (2004). Cellular and molecular regulation of muscle regeneration. Physiol Rev.

[9] Tafrihi M, Hasheminasab E (2019). MiRNAs: Biology, Biogenesis, their Web-based Tools, and Databases. Microrna.

[10] Saliminejad K, Khorram Khorshid HR, Soleymani Fard S, Ghaffari SH (2019). An overview of microRNAs: Biology, functions, therapeutics, and analysis methods. J Cell Physiol.

[11] Jing J, Jiang X, Zhu C (2021). Dynamic changes of miRNAs in skeletal muscle development at New Zealand rabbits. BMC Genomics.

[12] Wang H, Zhang Q, Wang B (2018). miR-22 regulates C2C12 myoblast proliferation and differentiation by targeting TGFBR1. Eur J Cell Biol.

[13] Guo J, Zhao W, Zhan S (2016). Identification and Expression Profiling of miRNAome in Goat longissimus dorsi Muscle from Prenatal Stages to a Neonatal Stage. PLoS ONE.

[14] Garros RF, Paul R, Connolly M (2017). MicroRNA-542 Promotes Mitochondrial Dysfunction and SMAD Activity and Is Elevated in Intensive Care Unit-acquired Weakness. Am J Respir Crit Care Med.

[15] Farre-Garros R, Lee JY, Natanek SA (2019). Quadriceps miR-542–3p and -5p are elevated in COPD and reduce function by inhibiting ribosomal and protein synthesis. J Appl Physiol (1985).

[16] Qin L, Chen Y, Liu X (2013). Integrative analysis of porcine microRNAome during skeletal muscle development. PLoS ONE.

[17] Shan T, Liu J, Xu Z, Wang Y (2019). Roles of phosphatase and tensin homolog in skeletal muscle. J Cell Physiol.

[18] Cai R, Zhang Q, Wang Y, Yong W, Zhao R, Pang W (2021). Lnc-ORA interacts with microRNA-532-3p and IGF2BP2 to inhibit skeletal muscle myogenesis. J Biol Chem.

[19] Yue F, Song C, Huang D (2021). PTEN Inhibition Ameliorates Muscle Degeneration and Improves Muscle Function in a Mouse Model of Duchenne Muscular Dystrophy. Mol Ther.

[20] Shen J, Wang J, Zhen H (2022). MicroRNA-381 Regulates Proliferation and Differentiation of Caprine Skeletal Muscle Satellite Cells by Targeting PTEN and JAG2. Int J Mol Sci.

[21] Mo M, Xiao Y, Huang S (2017). MicroRNA expressing profiles in A53T mutant alpha-synuclein transgenic mice and Parkinsonian. Oncotarget.

[22] Kabekkodu SP, Shukla V, Varghese VK, D' Souza J, Chakrabarty S, Satyamoorthy K (2018). Clustered miRNAs and their role in biological functions and diseases. Biol Rev Camb Philos Soc.

[23] Mishra S, Yadav T, Rani V (2016). Exploring miRNA based approaches in cancer diagnostics and therapeutics. Crit Rev Oncol Hematol.

[24] Ji H, Wang H, Ji Q (2020). Differential expression profile of microRNA in yak skeletal muscle and adipose tissue during development. Genes Genomics.

[25] Wang F, Chen X, Yu X, Lin Q (2019). Degradation of CCNB1 mediated by APC11 through UBA52 ubiquitination promotes cell cycle progression and proliferation of non-small cell lung cancer cells. Am J Transl Res.

[26] Spencer SL, Cappell SD, Tsai FC, Overton KW, Wang CL, Meyer T (2013). The proliferation-quiescence decision is controlled by a bifurcation in CDK2 activity at mitotic exit. Cell.

[27] Zang Y, Li J, Wan B, Tai Y (2020). circRNA circ-CCND1 promotes the proliferation of laryngeal squamous cell carcinoma through elevating CCND1 expression via interacting with HuR and miR-646. J Cell Mol Med.

[28] Ma Y, Shan Z, Liu Y (2021). CircTHBS1 targeting miR-211/CCND2 pathway to promote cell proliferation and migration potential in primary cystitis glandularis cells. Biosci Rep.

[29] Assi T, Kattan J, Rassy E (2020). Targeting CDK4 (cyclin-dependent kinase) amplification in liposarcoma: A comprehensive review. Crit Rev Oncol Hematol.

[30] Hong B, van den Heuvel AP, Prabhu VV, Zhang S, El-Deiry WS (2014). Targeting tumor suppressor p53 for cancer therapy: strategies, challenges and opportunities. Curr Drug Targets.

[31] Yuan L, Yuan P, Yuan H (2017). miR-542-3p inhibits colorectal cancer cell proliferation, migration and invasion by targeting OTUB1. Am J Cancer Res.

[32] Wei Q, Guo Z, Chen D, Jia X (2020). MiR-542-3p Suppresses Neuroblastoma Cell Proliferation and Invasion by Downregulation of KDM1A and ZNF346. Open Life Sci.

[33] Li J, Shao W, Feng H (2019). MiR-542-3p, a microRNA targeting CDK14, suppresses cell proliferation, invasiveness, and tumorigenesis of epithelial ovarian cancer. Biomed Pharmacother.

[34] Kureel J, Dixit M, Tyagi AM (2014). miR-542-3p suppresses osteoblast cell proliferation and differentiation, targets BMP-7 signaling and inhibits bone formation. Cell Death Dis.

[35] He Y, Zhang L, Cheng G (2017). Upregulation of circulating miR-21 is associated with poor prognosis of nasopharyngeal carcinoma. Int J Clin Exp Pathol.

[36] Wang Y, Huang JW, Castella M, Huntsman DG, Taniguchi T (2014). p53 is positively regulated by miR-542-3p. Cancer Res.

[37] Huang G, Zhou H, Xiang Q (2022). Exponential and efficient target-catalyst rolling circle amplification for label-free and ultrasensitive fluorescent detection of miR-21 and p53 gene. Anal Chim Acta.

[38] Wei X, You X, Zhang J, Zhou C (2019). c[RGDyk]-coated liposomes loaded with adriamycin and miR-21 mimics inhibit the growth of hepatoma cell line SMCC-7721 via up-regulating Bax and p53. Transl Cancer Res.

[39] Huang ZP, Espinoza-Lewis R, Wang DZ (2012). Determination of miRNA targets in skeletal muscle cells. Methods Mol Biol.

[40] Garcia-Perez I, Molsosa-Solanas A, Perello-Amoros M (2022). The Emerging Role of Long Non-Coding RNAs in Development and Function of Gilthead Sea Bream (Sparus aurata) Fast Skeletal Muscle. Cells.

[41] Xu M, Chen X, Chen D (2018). MicroRNA-499-5p regulates skeletal myofiber specification via NFATc1/MEF2C pathway and Thrap1/MEF2C axis. Life Sci.

[42] Sun Y, Wang H, Li Y, Liu S, Chen J, Ying H (2018). miR-24 and miR-122 Negatively Regulate the Transforming Growth Factor-beta/Smad Signaling Pathway in Skeletal Muscle Fibrosis. Mol Ther Nucleic Acids.

[43] Dey BK, Gagan J, Yan Z, Dutta A (2012). miR-26a is required for skeletal muscle differentiation and regeneration in mice. Genes Dev.

[44] Ling YH, Sui MH, Zheng Q (2018). miR-27b regulates myogenic proliferation and differentiation by targeting Pax3 in goat. Sci Rep.

[45] da Silva Diniz WJ, Banerjee P, Mazzoni G (2020). Interplay among miR-29 family, mineral metabolism, and gene regulation in Bos indicus muscle. Mol Genet Genomics.

[46] Honardoost M, Soleimani M, Arefian E, Sarookhani MR (2015). Expression Change of miR-214 and miR-135 during Muscle Differentiation. Cell J.

[47] Wang R, Kumar B, Doud EH (2022). Skeletal muscle-specific overexpression of miR-486 limits mammary tumor-induced skeletal muscle functional limitations. Mol Ther Nucleic Acids.

[48] Liu J, Liang X, Zhou D (2016). Coupling of mitochondrial function and skeletal muscle fiber type by a miR-499/Fnip1/AMPK circuit. EMBO Mol Med.

[49] Song C, Wang J, Ma Y (2018). Linc-smad7 promotes myoblast differentiation and muscle regeneration via sponging miR-125b. Epigenetics.

[50] Rivas DA, Peng F, Benard T, da Silva Ramos AS, Fielding RA, Margolis LM (2021). miR-19b-3p is associated with a diametric response to resistance exercise in older adults and regulates skeletal muscle anabolism via PTEN inhibition. Am J Physiol Cell Physiol.

[51] Li DM, Sun H (1997). TEP1, encoded by a candidate tumor suppressor locus, is a novel protein tyrosine phosphatase regulated by transforming growth factor beta. Cancer Res.

[52] Li J, Yen C, Liaw D (1997). PTEN, a putative protein tyrosine phosphatase gene mutated in human brain, breast, and prostate cancer. Science.

[53] Liaw D, Marsh DJ, Li J (1997). Germline mutations of the PTEN gene in Cowden disease, an inherited breast and thyroid cancer syndrome. Nat Genet.

[54] Hong L, Lai M, Chen M (2010). The miR-17-92 cluster of microRNAs confers tumorigenicity by inhibiting oncogene-induced senescence. Cancer Res.

[55] Lujambio A, Lowe SW (2012). The microcosmos of cancer. Nature.

[56] Liu HY, Zhang YY, Zhu BL (2019). miR-21 regulates the proliferation and apoptosis of ovarian cancer cells through PTEN/PI3K/AKT. Eur Rev Med Pharmacol Sci.

[57] Yue Y, Feng X, Jia Y (2023). miR-424(322)-5p targets Ezh1 to inhibit the proliferation and differentiation of myoblasts. Acta Biochim Biophys Sin (Shanghai).

[58] Fischer-Lougheed J, Liu JH, Espinos E (2001). Human myoblast fusion requires expression of functional inward rectifier Kir2.1 channels. J Cell Biol.

