# Supplementary material for: MicroRNA-542-3p targets Pten to inhibit the myoblasts proliferation but suppresses myogenic differentiation independent of targeted Pten
Source: BMC Genomics. 2024 Apr 1;25:325. doi: 10.1186/s12864-024-10260-y (PMC10983626; doi:10.1186/s12864-024-10260-y)
Supplement: Supplementary file 1 — Supplementary Material 1. [file 12864_2024_10260_MOESM1_ESM.docx]

Supplementary Information

**MicroRNA-542-3p targets *Pten* to inhibit the myoblasts proliferation but suppresses myogenic differentiation independent of targeted *Pten***

**Dandan Li^1,2,3↑^,** **Yongqi Yue^4↑^, Xinxin Feng^1,5^, Weibing Lv^1,2,3^, Yilin Fan^1,2,3^, Peiran Sha^1,2,3^, Te Zhao^1,2,3^, Yaqiu Lin^1,2,3^, Xianrong Xiong^1,2,3^, Jian Li^1,2,3^, and** **Yan Xiong^1,2,3,*^**

^1^Key Laboratory of Qinghai-Tibetan Plateau Animal Genetic Resource Reservation and Utilization, Ministry of Education/Sichuan Province, Southwest Minzu University, Chengdu, 610041, China, ^2^College of Animal & Veterinary Sciences, Southwest Minzu University, Chengdu, 610041, China, ^3^Key Laboratory of Animal Science of National Ethnic Affairs Commission of China, Southwest Minzu University, Chengdu, 610041, China, ^4^College of Animal Science and Technology, Northwest A&F University, Shaanxi, 712100, China, ^5^Chongxin county animal husbandry and veterinary center, Pingliang, 744200, China.

^↑^Dandan Li and Yongqi Yue contributed equally to this work.

^*^Correspondence: Yan Xiong: [xiongyan0910@126.com](mailto:xiongyan0910@126.com)


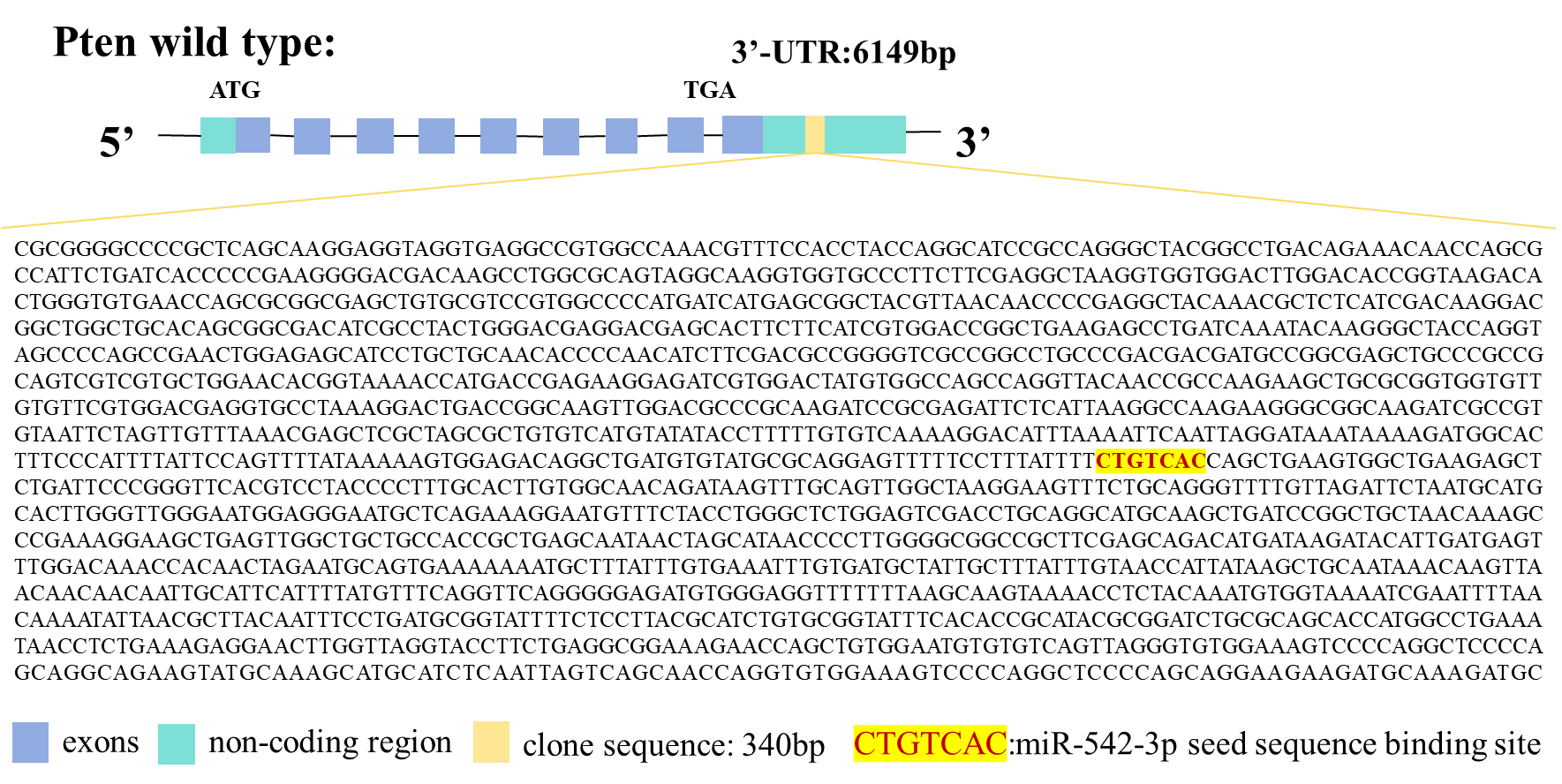


**Supplementary Fig. S1.** Site of binding to miR-542-3p seed sequence in a wild-type 3’-UTR clone sequence of Pten.


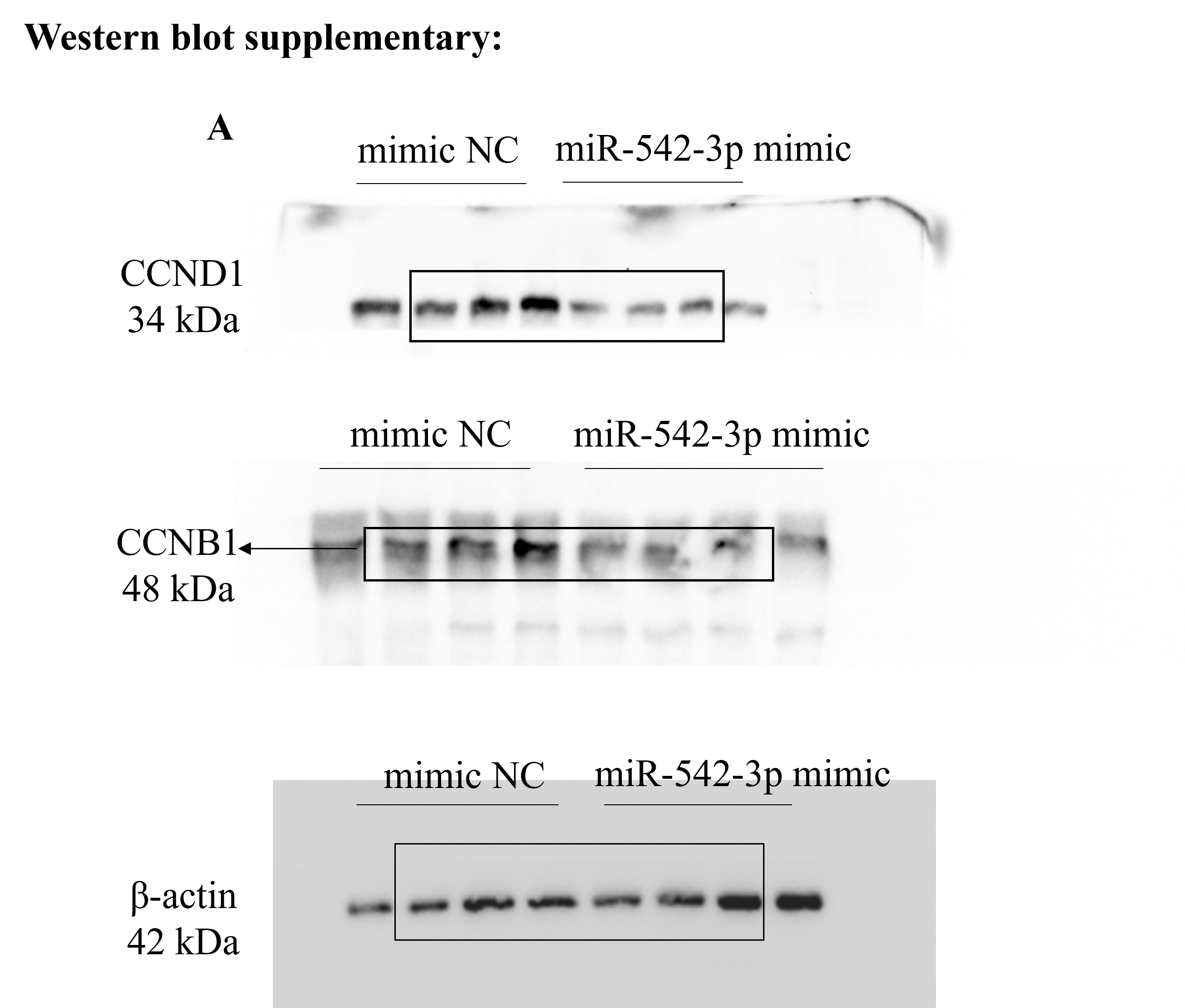


**Supplementary Fig. S2.** miR-542-3p inhibits myoblast proliferation. **A** The full-length blots of CCND1, CCNB1, and β-actin proteins after overexpression of miR-542-3p. 4 biological replicates were detected, 3 of which were cut shown in Figure 2C.


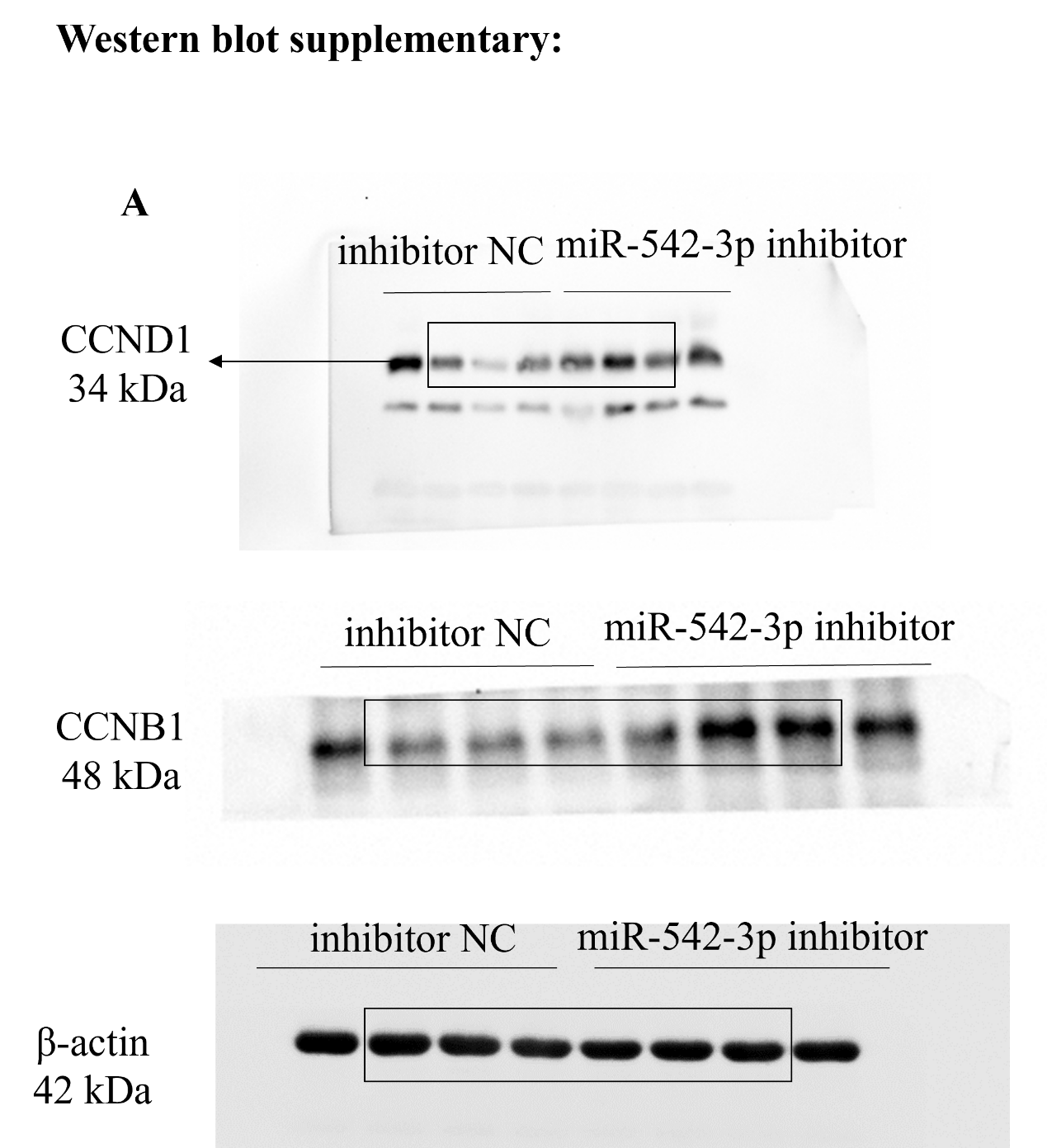


**Supplementary Fig. S3.** miR-542-3p inhibits myoblast proliferation. **A** The full-length blots of CCND1, CCNB1, and β-actin proteins after inhibition of miR-542-3p. 4 biological replicates were detected, 3 of which were cut shown in Figure 3C.


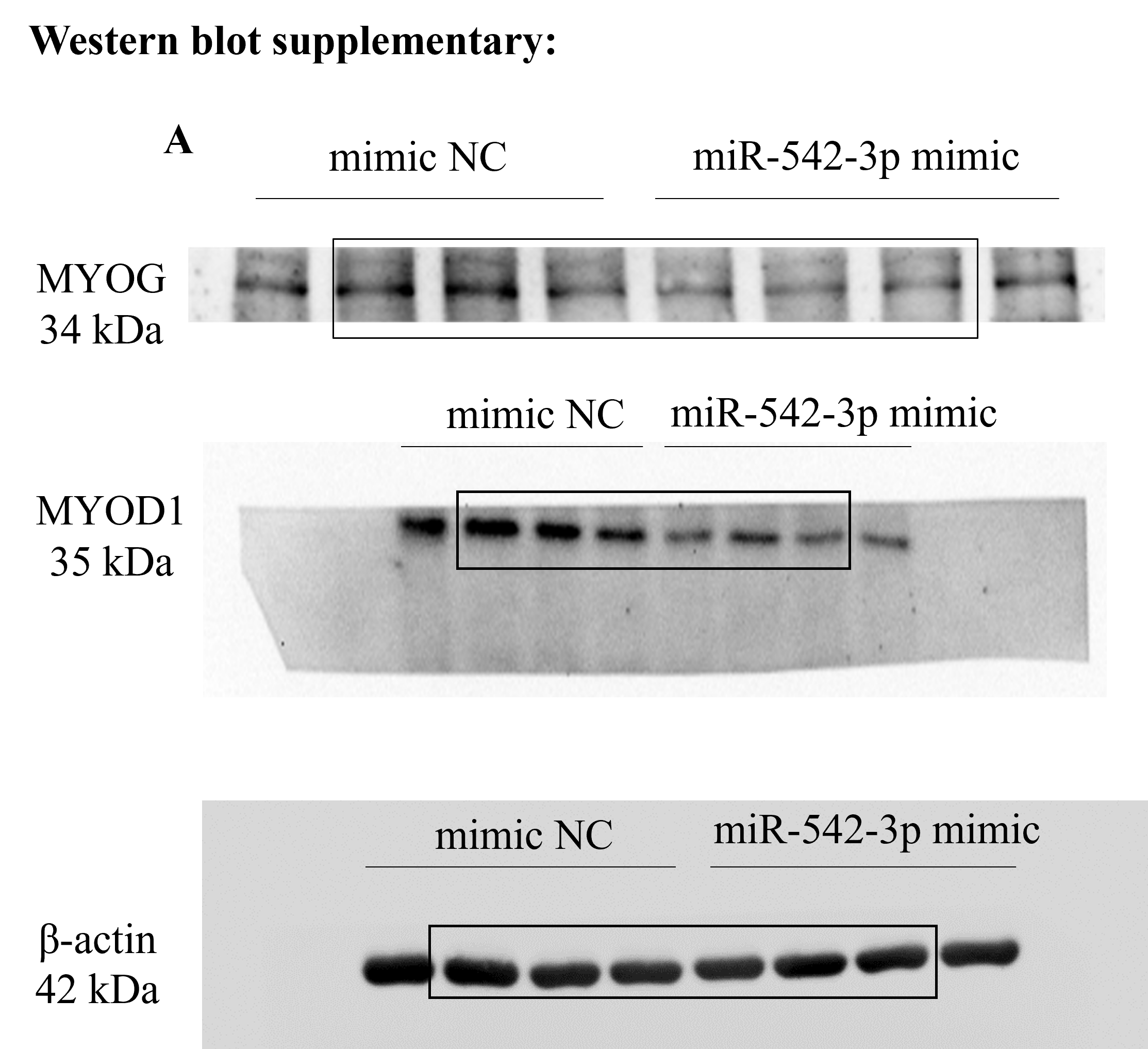


**Supplementary Fig. S4.** miR-542-3p inhibits myogenic differentiation. **A** The full-length blots of MYOG, MYOD1, and β-actin proteins after overexpression of miR-542-3p. 4 biological replicates were detected, 3 of which were cut shown in Figure 4B.


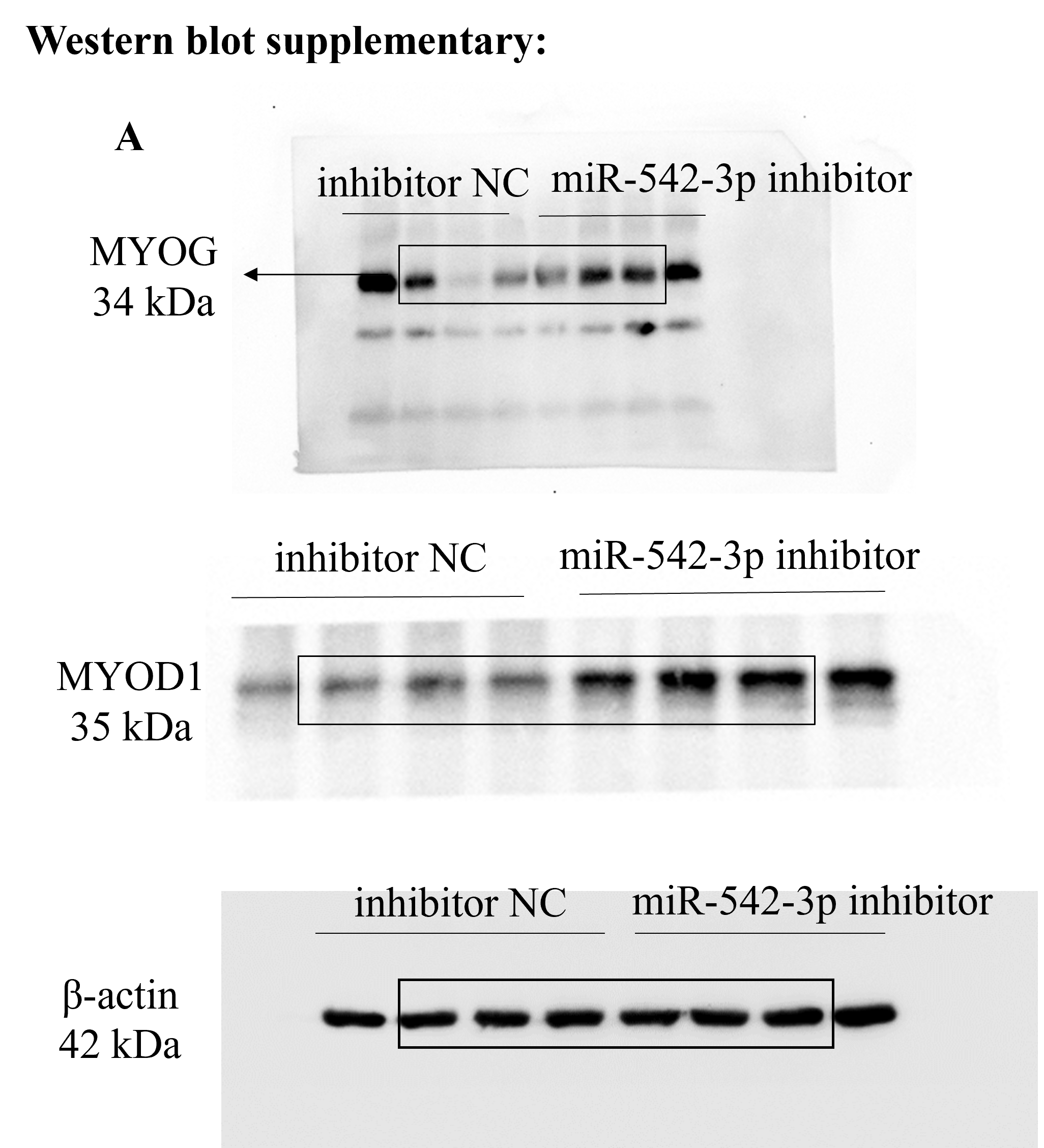


**Supplementary Fig. S5.** miR-542-3p inhibits myogenic differentiation. **A** The full-length blots of MYOG, MYOD1, and β-actin proteins after inhibition of miR-542-3p. 4 biological replicates were detected, 3 of which were cut shown in Figure 5B.


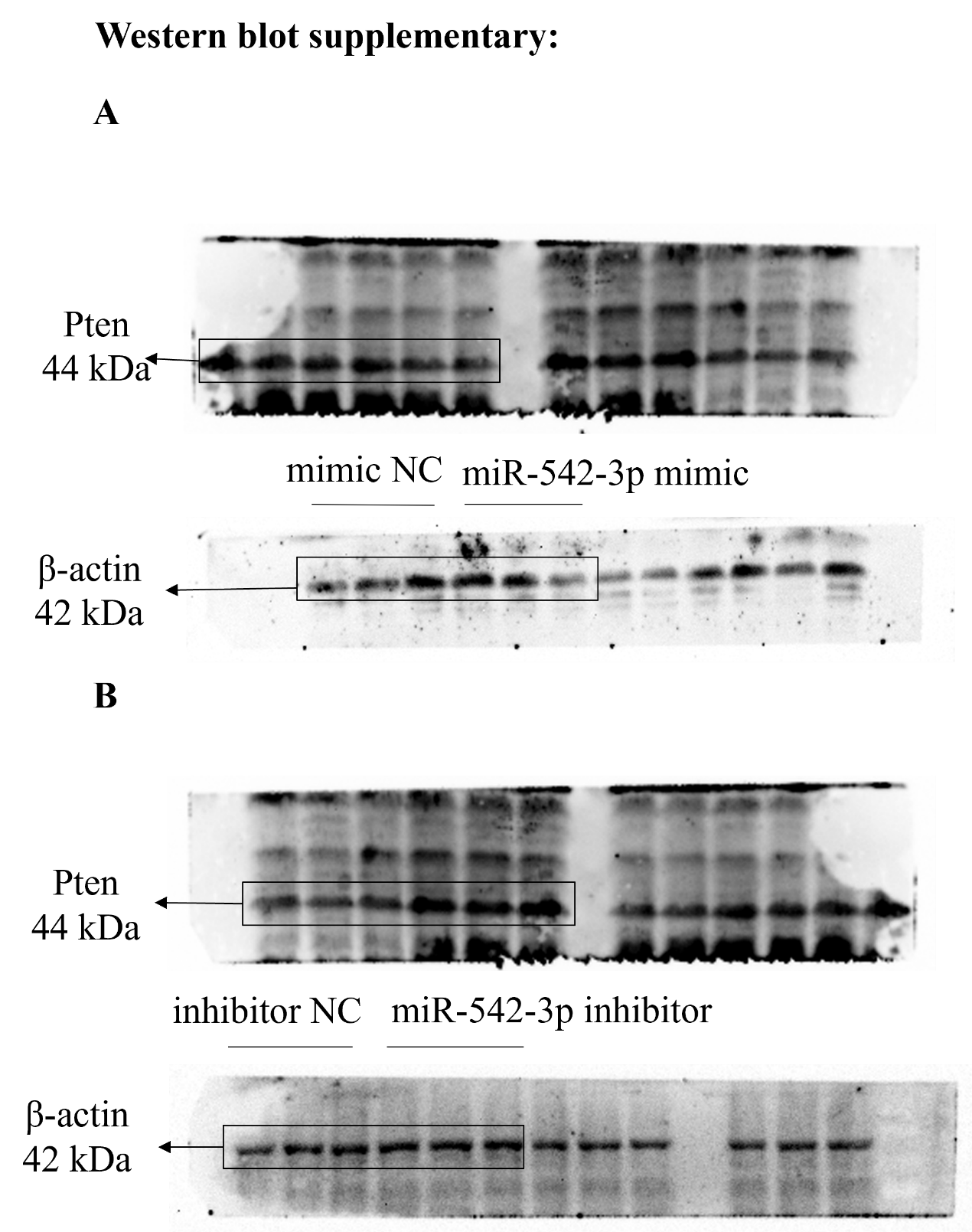


**Supplementary Fig. S6.** miR-542-3p directly targets the Pten gene. **A** The full-length blots of Pten and β-actin proteins after overexpression of miR-542-3p were cut shown in Figure 6G. **B** The full-length blots of Pten and β-actin proteins after inhibition of miR-542-3p were cut shown in Figure 6I.
